# The Role of Macrophage Migration Inhibitory Factor in the Function of Intestinal Barrier

**DOI:** 10.1038/s41598-018-24706-3

**Published:** 2018-04-20

**Authors:** Milica Vujicic, Tamara Saksida, Sanja Despotovic, Svetlana Sokovic Bajic, Ivana Lalić, Ivan Koprivica, Dragica Gajic, Natasa Golic, Maja Tolinacki, Ivana Stojanovic

**Affiliations:** 1 Institute for Biological Research “Sinisa Stankovic”, University of Belgrade, Department of Immunology, Belgrade, 11060 Serbia; 2 Faculty of Medicine, University of Belgrade, Institute of Histology and Embryology, Belgrade, 11000 Serbia; 3 Institute of Molecular Genetics and Genetic Engineering (IMGGE), University of Belgrade, Belgrade, 11000 Serbia

## Abstract

Macrophage migration inhibitory factor (MIF) is a multifunctional protein that is involved in the development of gut-related inflammation. To investigate the role of MIF in the function of the intestinal barrier, we have explored intestinal permeability and gut-associated immune response in MIF-deficient (MIF-KO) mice. The absence of MIF provoked impairment of tight and adherens epithelial junctions in the colon through the disturbance of E-cadherin, zonula occludens-1, occludin and claudin-2 expression, which lead to the increase of intestinal barrier permeability. In these circumstances the diversity and content of gut microbiota in MIF-KO mice was considerably different compared to wild type mice. This change in microbiota was accompanied by an increased intestinal IgA concentration and a higher production of pro-inflammatory cytokines TNF and IFN-γ in mesenteric lymph nodes of MIF-KO mice. The forced changes of microbiota executed by antibiotics prevented the “leakage” of the barrier in MIF-KO mice, probably through up-regulation of occludin expression and normalization of cellular pore diameters. In addition, cytokine secretion was normalized after the treatment with antibiotics. These results suggest that MIF participates in the maintenance of physiological microbiota diversity and immunosurveillance, which in turn enables the proper intestinal barrier function.

## Introduction

Functional intestinal barrier is crucial for maintaining homeostasis between the symbiotic microbiota in the lumen of the gut and the rest of the organism. Intestinal barrier is composed of a physical barrier made of epithelial cells tightly connected by tight and adhesive junctions, as well as a functional barrier made of immune cell- or epithelial cell-secreted mediators that control both diversity and number of microbiota populations^1^. A breach in the barrier is usually prevented by cells of the gut-associated lymphoid tissue (GALT) that perform immune surveillance of the gut-related microbiota. Increasing evidence implies the link between the dysfunctional intestinal barrier and development of several pathogenic conditions such as autoinflammatory and autoimmune disease, obesity, neuroinflammation and so on^2,3^.

One of the molecules that is implicated in the development of various diseases with inflammatory background and can potentially regulate the function of the intestinal barrier is macrophage migration inhibitory factor (MIF). This protein can be secreted both from immune and non-immune cells including gut epithelial cells^4^. Its primary function is to stimulate the retention of macrophages at the site of inflammation and thus facilitate the eradication of the pathogen^5^. This role of MIF might be considered detrimental in the circumstances of, for example, ulcerative colitis. Namely, MIF is highly expressed in the colon of mice and it probably mediates colitis development through the stimulation of macrophage infiltration in the colon^6–8^.

There is no experimental evidence for the possible influence of MIF on the gut microbiota. Having in mind its functions, MIF might play a dual role in the immunosurveillance of microbiota. On the one hand, MIF can directly stimulate immune cells to recognize bacteria^5^, while on the other hand MIF can enable proper transport of antigens from the lumen of the gut through microfold cells (interspersed in the epithelium and located next to Peyer’s patches) to the immune cells scattered in the lamina propria or situated within more organized structures such as Peyer’s patches or mesenteric lymph nodes (MLN)^9,10^. Although there is evidence that MIF is involved in the colitis pathogenesis, whose hallmark is high intestinal permeability^8^, there is no available data on the possible role of MIF in the physiology of intestinal barrier in disease-free animals. In order to investigate this, mice with genetic MIF deletion (MIF-KO) were used and the effect of MIF absence was determined both at the level of gut epithelial cells and at the level of GALT-related immune response.

## Results and Discussions

### MIF absence promotes intestinal permeability in the colon

**Figure 1:**
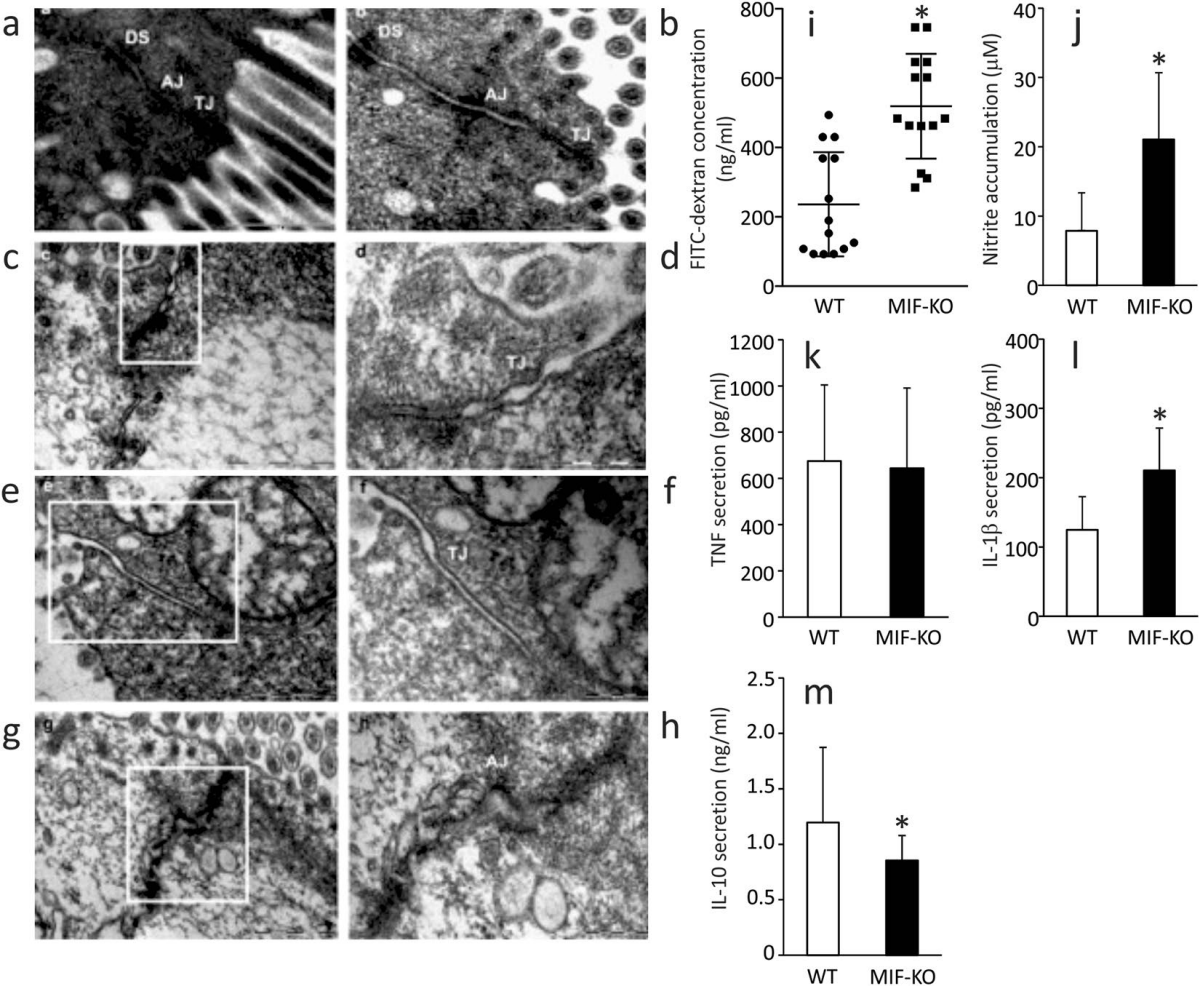
The influence of MIF on the intestinal permeability. (**a**–**h**) Electron micrographs showing the tight junctions (TJ), adherens junctions (AJ) and desmosomes (DS) of colon epithelial cells. (**a,b**) TJ, AJ and DS in the colon of WT mice (x56000). (**c**) TJ in the colon of MIF-KO animals were wider, without complete obliteration of intercellular space (x56000). (**d**) The same TJ in MIF-KO animal, on higher magnification (x140000). (**e**) Wide TJ in the colon of MIF-KO animal; obliteration of intercellular space is missing (x56000). (**f**) The same TJ in MIF-KO animal, on higher magnification (x89000). (**g**) Wide AJ in the colon of MIF-KO animal (x44000). (**h**) The same AJ in MIF-KO animal, on higher magnification (x89000). (**i**) Concentration of serum FITC-dextran representing intestinal permeability. (**j**) Nitric oxide secretion from the peritoneal cells stimulated *in vitro* by LPS. (**k–m**) Cytokine secretion from peritoneal cells stimulated with LPS *in vitro*. For statistical analysis, test of normality and Mann–Whitney U-test were used. *p<0.05 represents the significant difference between values of WT vs MIF-KO mice.

In order to visualize the epithelial layer of the large intestine, transmission electron microscopy was employed. Enterocytes of MIF-KO and wild type (WT) mice had comparable appearance: microvilli were well developed; cells were full of mitochondria and vesicles. The junctional complex was clearly visible at the luminal side of cell-cell contact: tight junctions were located most apically, followed from the apical to the basal side of the cell by the adherens junctions and desmosomes (Fig. 1a,b). In the colon of WT animals the tight junctions were narrow with the appearance of a series of fusions (“kissing points”), involving the outer leaflets of the plasma membranes of adjacent cells. At the kissing points, the intercellular spaces were completely “locked” and a characteristic pentalaminar structure of tight junctions could be observed (Fig. 1a,b). The cytoplasmic side of adherens junctions was associated with actin filaments from the terminal network (Fig. 1b). In contrast, in the colon of MIF-KO mice most of the tight junctions were less well-defined, the “kissing points” were less frequently seen and the complete obliteration of intercellular spaces was missing (Fig. 1c,e,f). Adherens junctions were well developed but wider in comparison to the ones seen in WT animals (Fig. 1g,h). Morphometric analysis confirmed our observations: the width of the tight and adherens junctions was considerably increased in the colon of MIF-KO animals compared with WT animals (Table 1). In the colon of both WT and MIF-KO animals desmosomes were frequently seen with normal appearance as densely stained plaques on the intercellular face of the membranes, with a prominent mid-line (Fig. 1a–c). Further experiments confirmed the microscopy data, since MIF-KO mice had a higher concentration of FITC-dextran in the serum after per os administration (Fig. 1i). These results suggest that MIF absence provoked an increase in intestinal permeability.

**Table 1:** Width of tight and adherens junction in the colon epithelial cells.

Width (nm)	WT median (IQR)^&^	WT+Abx median (IQR^)&^	MIF-KO median (IQR)^&^	MIF-KO+Abx median (IQR)&
Tight junctions	0.003 (0.002–0.004)	0.003 (0.002–0.005)	0.006 (0.004–0.010)^*^	0.003 (0.002–0.004)^#^
Adherens junctions	0.015 (0.012–0.020)	0.016 (0.011–0.021)	0.024 (0.017–0.039)^*^	0.016 (0.011–0.022)^#^

Abx – antibiotics;

^*^p<0.001 represent the significant difference between WT and MIF-KO;

^#^p<0.001 represent the significant difference between MIF-KO and MIF-KO+Abx;

^&^The interquartile range (IQR);

For statistical analysis the Student's t-test was used to compare mean values between two groups and 50 junctions per mouse were counted (number of mice per group 12).

Although epithelial connections were disturbed in the colon of MIF-KO mice, there were no differences in any measured elements of intestinal mucosa (thickness of the layers, height of the epithelial layer, crypt morphology, number of cells in the lamina propria) between MIF-KO and WT animals (Supplemental data and Figure S1, Table S1).

Another proof of the “leaky” gut in MIF-KO mice is the activated state of macrophages within the peritoneum^11^. It is suggested that if the barrier is permeable, bacteria from the gut can migrate to distant sites in the organism and activate resident macrophages, as it is in the case of the peritoneum. Although the number of isolated peritoneal cells was the same in both strains (7.9 ± 3.8 × 10^6^ vs 6.3 ± 2.9 × 10^6^, WT vs MIF-KO, respectively), macrophages from MIF-KO mice produced higher levels of nitric oxide (Fig. 1j), equal TNF levels (Fig. 1k), higher levels of IL-1β (Fig. 1l), and lower levels of IL-10 (Fig. 1m), suggesting that they were exposed to the bacterial species that might have migrated from the gut. Our results are in contrast with previous data obtained in dextran sodium sulfate (DSS)-treated MIF-KO mice. In this colitis model, the absence of MIF prevents the development of colon histopathological changes induced by DSS^8^. However, the permeability was not monitored in these mice and they were bred on a different genetic background.

### The effect of MIF absence on the intestinal epithelial cells

**Figure 2:**
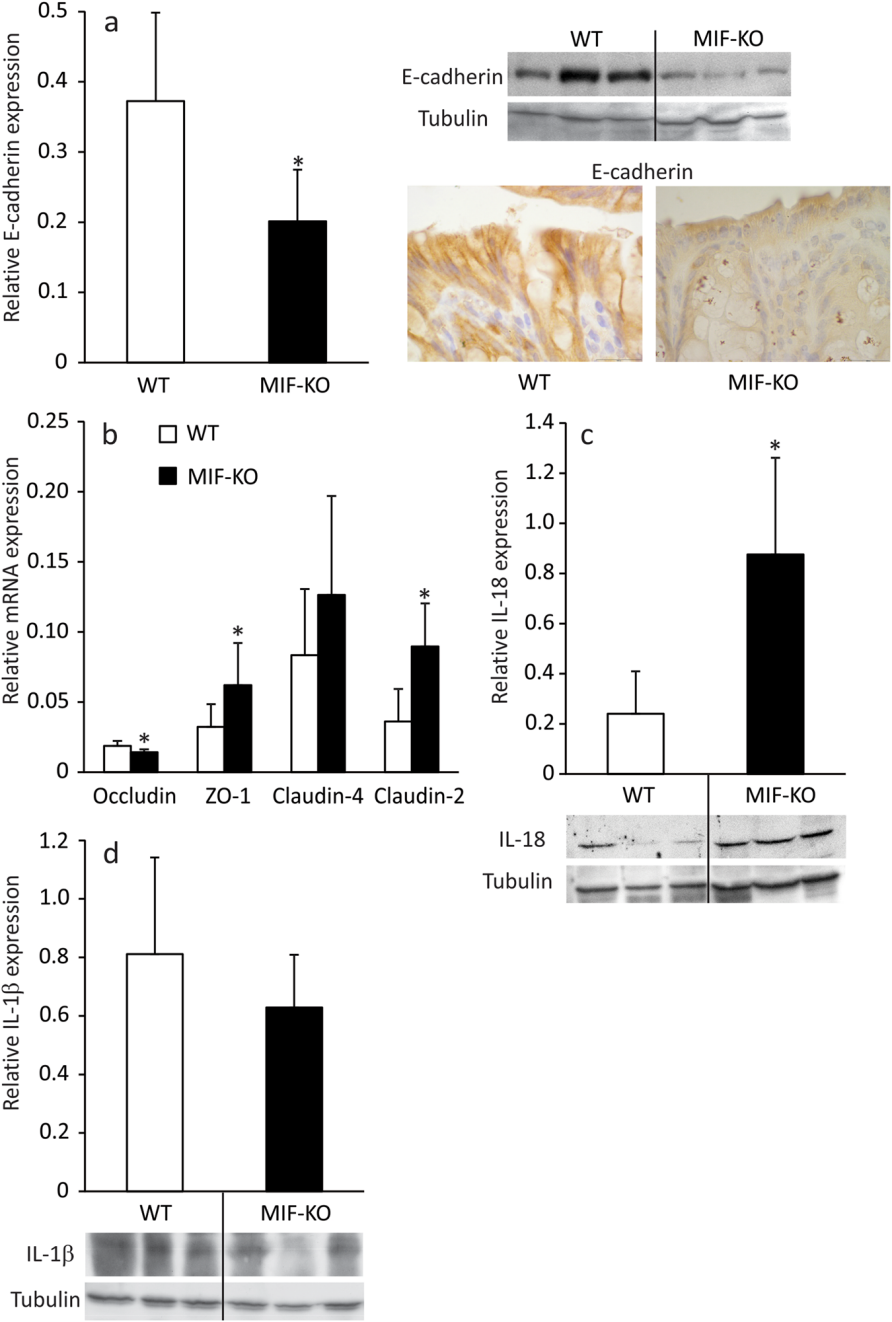
The effect of MIF absence on colon epithelial cells. The expression of E-cadherin (**a**), relative to tubulin expression was measured by western blot in epithelial cells of the large intestine. Representative blot is shown on the right-hand side along with immunostaining for E-cadherin of colon sections. The mRNA expression of tight junction proteins (**b**) was determined by real-time PCR and normalized to β-actin expression. IL-18 (**c**) and IL-1β (**d**) protein expression was measured by western blot as in (**a**). Representative blot images are shown below the graphs. Presented results are average ± SD from 16 animals per group, obtained in 3 separate experiments. For statistical analysis, test of normality and Mann–Whitney U-test were used. *p<0.05 represents the significant difference between values of protein and mRNA expression in epithelial cells of WT vs MIF-KO mice.

Although MIF seems to be of the outmost importance for the survival of colon epithelial cells^12^, there is no data on the possible role of MIF in the maintenance of the cell-cell junctions. Since the “leakage” of the gut is often due to the irregular infrastructure of epithelial cells junctions^13^, the content of E-cadherin and expression of occludin, pore-forming and junction-forming claudins and zonula occludens-1 (ZO-1) in the colon epithelial cells was examined. E-cadherin is responsible for the proper architecture of adherens junctions^14^ and its considerably lower content detected by western blot and immunohistochemically in MIF-KO epithelial cells (Fig. 2a) coincided with already observed wider adherens junctions. Formation of tight junction depends upon complex interactions between occludin, claudins and ZO-1^15^. Lower occludin mRNA expression (Fig. 2b) could be responsible for wider tight junctions in MIF-KO mice. Although the expression of ZO-1, a scaffold protein that is involved in the linkage of junction proteins to actin filaments^15^, is increased in MIF-KO epithelial cells, a higher expression of claudin-2 (Fig. 2b), which is involved in pore formation^16^, could further attribute to wider pores seen in between epithelial cells in the absence of MIF. Finally, there was no significant difference between WT and MIF-KO in the expression of claudin-4, a member of the tight junction complex that regulates the tightness of the gap between epithelial cells^16^.

It is already known that epithelial junctions can be disturbed through the influence of IL-1β and IL-18^17,18^. Consistent with the impaired morphology of epithelial junctions, MIF-KO epithelial cells of the large intestine produced higher levels of IL-18 compared to WT (Fig. 2c). However, IL-1β production was comparable between the strains (Fig. 2d). Since IL-18 is an end product of activation of microbial receptors^19^, the observed up-regulation of IL-18 might be a consequence of microbiota migration through a permeable intestinal barrier. Also, IL-18 is an inducer of IFN-γ production in lymphocytes^20^ and this up-regulation in epithelial cells could serve to potentiate an adaptive immune response in the GALT.

### The influence of MIF absence on immune surveillance in GALT

**Figure 3:**
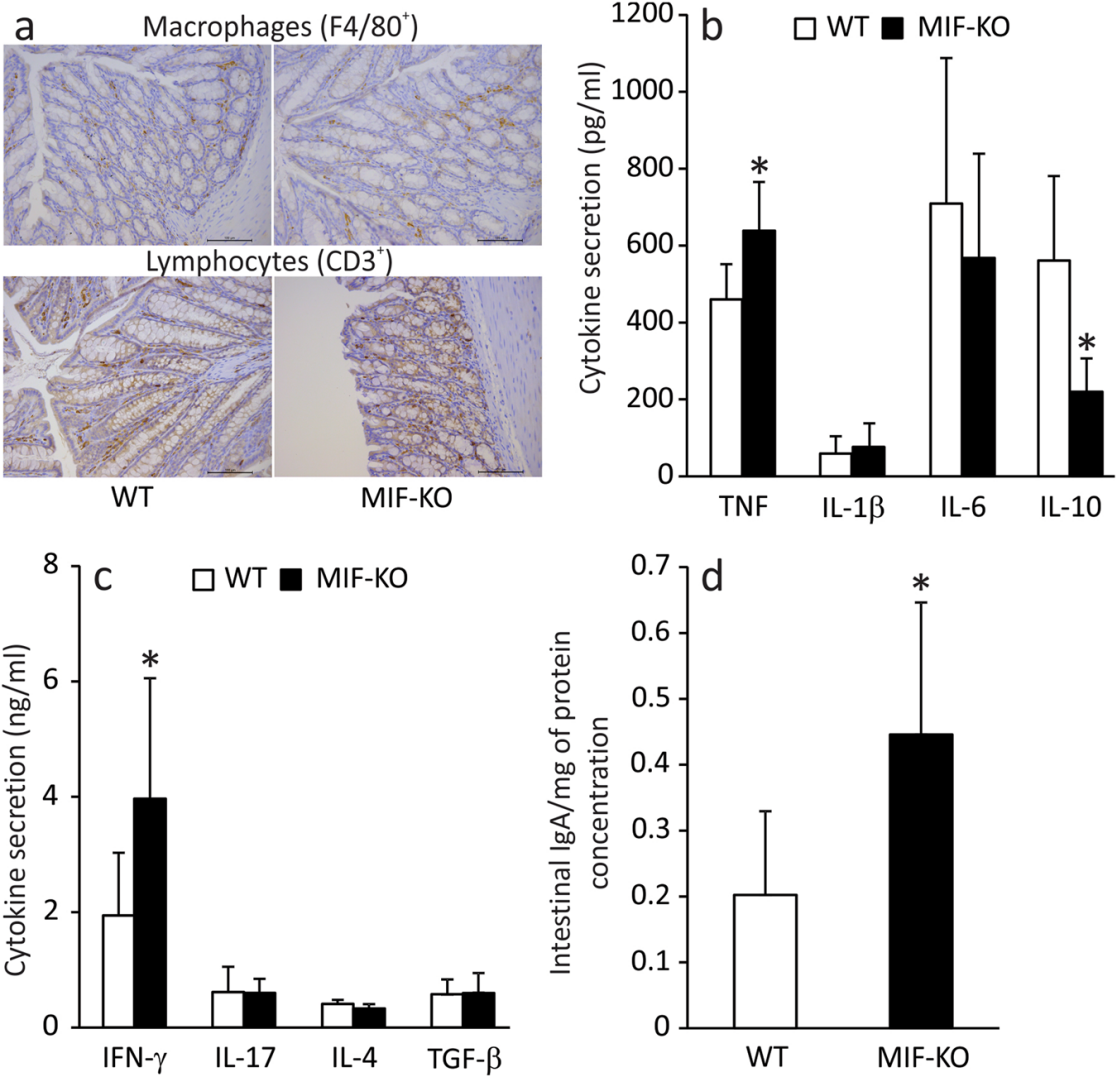
The effect of MIF absence on the cytokine and IgA secretion within GALT. F4/80^+^ macrophages and CD3^+^ lymphocytes in immunohistochemical sections of colon lamina propria. At least 15 sections per mouse were analysed in a blinded fashion (**a**). Cytokine secretion was measured in MLN cells after 24 h of either LPS stimulation (**b**), or ConA stimulation (**c**). Intestinal IgA content was measured by western blot and normalized to the whole protein concentration in the samples (**d**). Results were obtained from 16 animals per group gathered from 3 separate experiments. For statistical analysis, test of normality and Mann–Whitney U-test were used. *p<0.05 represents the significant difference between values of cytokine and IgA concentration from MLN of WT vs MIF-KO mice.

As already stated, colon GALT is comprised of intraepithelial lymphocytes, lymphocytes scattered within lamina propria and cells in mesenteric lymph nodes (MLN). Although intestinal permeability was considerably increased in the absence of MIF, the number of F4/80^+^ macrophages or CD3^+^ lymphocytes in the lamina propria did not differ between MIF-KO and WT mice (Table 2 and Fig. 3a). In addition, no differences were found in the number of MLN cells or their proliferative capacity (Table 3). Likewise, the proportions of cells of the innate immune system: macrophages (M1 - pro-inflammatory, or M2 - anti-inflammatory), dendritic cells and NK cells were equal between the strains (Table 3). Cells of the adaptive immune system: CD4^+^ helper lymphocytes, CD4^+^CD25^+^ T regulatory cells, CD8^+^ cytotoxic lymphocytes, B220^+^ B cells or CD5^+^CD19^+^IL-10^+^ B regulatory cells were also similarly distributed in MLN of WT and MIF-KO mice (Table 3). Although the immune cell distribution appeared normal, macrophage-related production of TNF was considerably increased in MIF-KO MLN cells stimulated *in vitro* (Fig. 3b). It has already been shown that TNF up-regulates claudin-2 expression in epithelial HT-29/B6 cells and thereby disturbs tight junction formation. This scenario could be operable in MIF-KO mice since we indeed observed increased claudin-2 expression in the epithelium^21^. In contrast, IL-1β and IL-6 remained the same as in MLN cells of WT mice (Fig. 3b). Interestingly, the inflammatory milieu of MLN in MIF-KO mice was potentiated by decreased production of anti-inflammatory cytokine IL-10 (Fig. 3b). The innate MIF function is to stimulate macrophage activation^5^ and MIF deficiency should interfere with macrophage functions. This is not the case in our study, most likely as a result of other cytokines taking over some of the functions of MIF and responding adequately to the bacterial stimuli from the gut. As for cytokines from the immune cells of adaptive immunity, it was determined that lymphocyte-mediated production of IFN-γ was up-regulated in the absence of MIF (Fig. 3c), suggesting that Th1 and CD8^+^ populations might mediate the adaptive response to the unwanted entrance of gut microbiota. This is in accordance with the previously observed high expression of IL-18 by epithelial cells, since IL-18 can drive Th1 immunity against bacterial infections^20^. Apart from an anti-bacterial effect, another outcome of elevated IFN-γ could be the observed disturbance of tight junctions. Recent studies suggest that IFN-γ increases paracellular permeability in intestinal epithelial cells through the redistribution and expression of tight junction proteins and the rearrangement of the actin cytoskeleton^22^. IL-17 and cytokines that represent the anti-inflammatory arm of the adaptive immunity - IL-4, TGF-β and IL-10 (produced in Breg cells) were equally produced from the MLN cells of MIF-KO and WT mice (Fig. 3c and Table 2). Finally, we have measured the intestinal content of IgA, an immunoglobulin produced by B lymphocytes and which controls the content of the microbiota^23^ and found that IgA levels were considerably increased in MIF-KO colon compared to WT (Fig. 3d).

**Table 2:** Macrophages and lymphocytes in colon lamina propria of WT and MIF-KO mice.

Cell number/0.1 mm^2^ of lamina propria	WT	WT+Abx	MIF-KO	MIF-KO+Abx
F4/80^+^ macrophages (mean ± SD)	91.98 ± 25.03	107.26 ± 15.93	103.55 ± 26.17	102.10 ± 33.55
CD3^+^ lymphocytes (mean ± SD)	96.10 ± 43.43	94.83 ± 35.10	98.64 ± 40.40	98.75 ± 35.45

Number of mice: 8 per group. At least 15 sections per mouse was analysed. For statistical difference ANOVA (posthoc Tukey) was used. For determination of the difference between two groups Student t test for two independent samples was used.

**Table 3:** The influence of MIF absence on cellularity, proliferation and immune cell composition in MLN.

MLN	WT	MIF-KO
Number of cells (x10^6^)	24.5 ± 8.7	25.3 ± 7.1
Proliferation index*	1.4 ± 0.1	1.5 ± 0.4
F4/80^+^CD40^+^ (M1) (%)	2.4 ± 0.6	3.0 ± 1.2
F4/80^+^CD206^+^ (M2) (%)	1.7 ± 0.6	2.3 ± 1.1
CD11c^+^ (DC) (%)	12.0 ± 1.4	13.2 ± 3.6
NK (%)	0.6 ± 0.4	0.5 ± 0.1
CD4^+^ (%)	28.2 ± 1.0	29.7 ± 2.6
CD4^+^CD25^high^ - Treg (%)	1.7 ± 0.1	1.6 ± 0.1
CD8^+^ (%)	25.2 ± 0.9	27.8 ± 7.0
B220^+^ (%)	41.1 ± 8.8	38.2 ± 3.3
CD5^+^CD19^+^IL-10^+^ - Breg (%)	11.0 ± 6.2	11.7 ± 2.6
IL-10+ total (%)	2.2 ± 0.1	2.6 ± 0.7

*Proliferation index was calculated when values obtained for ConA-stimulated cells were divided by values of non-treated cell cultures after 24 h of incubation; Treg – T regulatory cells; Breg – B regulatory cells.

Overall, these results suggest that the perturbation in the production of cytokines within GALT is a reaction to the observed breach in the intestinal barrier that occurred as a consequence of MIF absence. Although characterized by higher cytokine production, the reaction of MIF-deficient immune cells to the microbiota exposure seemed to be quite limited. This might be explained by the role of MIF in the sampling of antigens from the gut lumen^9^. Without proper antigen transport, dendritic cells located beneath the microfold cells that mediate the transport cannot optimally perform their function.

### MIF absence affects gut microbiota composition

**Figure 4:**
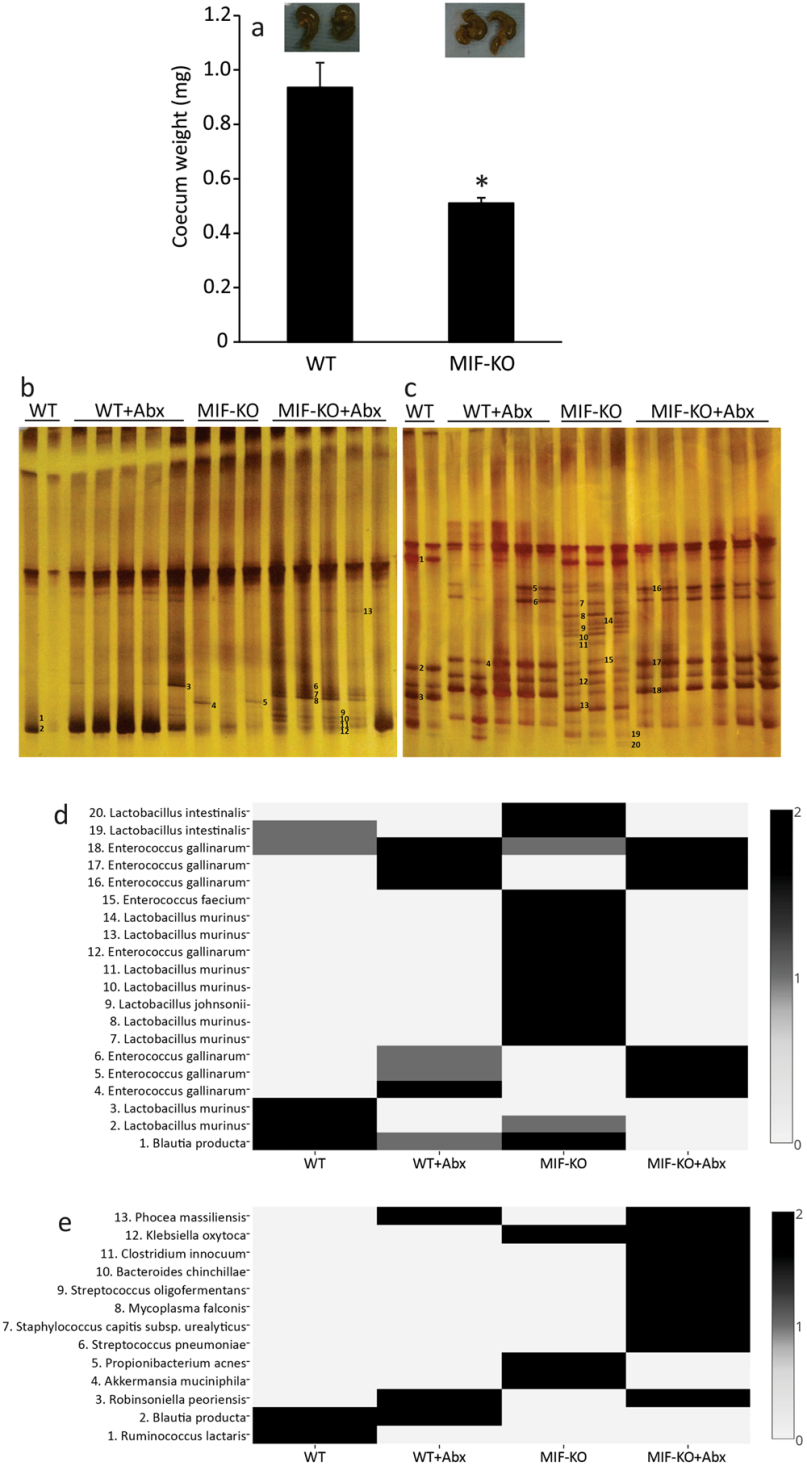
Comparison between microbiota contents in WT and MIF-KO mice. Caecum weight (**a**) with representative images above the graph. Denaturing gradient gel electrophoresis (DGGE) profiles of rDNA amplicons obtained using a universal primer set complementary to Eubacteria (**b**) and *Lactobacillus*-specific primer set (**c**) on bacterial DNA isolated from faecal samples. Bands indicated by numbers (1–13 for universal primers and 1–20 for *Lactobacillus*-specific primers) were excised, cloned and sequenced. The identity of the bands is reported in the heat map for universal (**d**) and *Lactobacillus*-specific primers (**e**), where the numbers given at the left ordinate refer to the marked bands on DGGE gels. Results were approximated on the relative scale ranging from 0 (white) as the lowest values, progressing to 2 (black) as the highest values. Number of mice per group ranged from 13 to 17. For statistical analysis, test of normality and Student t-test were used. *p<0.05 represents the significant difference between caecum weight of WT vs MIF-KO mice.

There is a constant bi-directional interplay between the mucosal immune system and gut microbiota. Since there is evidence of intestinal leakage and limited perturbation in GALT-associated immune surveillance in MIF-KO mice, it is presumed that MIF absence is associated with the disturbance of gut microbiota composition. Therefore, the weight and content of caeca were measured, since this is the place where a collection of innate microbiota of rodents resides^24^. In addition to a change in the caecum weight which was lower in MIF-KO mice (Fig. 4a), a distinguished difference in the microbiota composition between WT and MIF-KO mice was observed (Fig. 4b,d), including higher diversity of Lactobacilli strains (Fig. 4c,e). Interestingly, *Ruminoccocus lactaris*, that is a normal constituent of mouse microbiota^25^, was completely absent in MIF-KO gut (Fig. 4e). Further, *Blautia producta*, an indicator of the healthy gut and accountable for digestion of complex carbohydrates, was more abundantly present in samples collected from WT than in MIF-KO mice (Fig. 4e). This was the case when a universal primer set was used (Fig. 4e), while the difference in *Blautia* levels between WT and MIF-KO mice was not detected with a Lactobacilli-specific primer set (Fig. 4d). Since higher *Blautia* levels are associated with the decrease of obesity in high-fat fed rats^26^, a possible correlation between *Blautia* levels and the fact that MIF-KO mice are prone to obesity development after 6 months of age^27^ needs to be further investigated. On the other hand, *Akkermansia muciniphila*, an intestinal symbiont considered a guardian of intestinal mucosa^28^, was only detected in MIF-KO (Fig. 4e). Although the exact bacterial species related to the increased intestinal leakage were not detected, the mere change in the ratio between certain microbiota species^29^ could account for the observed MIF-KO phenotype. The *Lactobacilaceae* species that inhabited the intestine of MIF-KO mice and not WT mice (specific strains from line 7 until 15 in Fig. 4e) likely emerged as a consequence of improper immune surveillance in GALT in the absence of MIF. Although the immune reaction in GALT manifested through the secretion of pro-inflammatory cytokines and IgA, it seems that MIF-KO mice were unable to properly control the gut microbiota content. In the immune response, MIF is generally involved in eradicating bacterial infections through up-regulation of toll-like receptor 4 or MHC class II on macrophages^5^, so the absence of MIF might impair these macrophage functions.

### How does MIF regulate the function of the intestinal barrier?

**Figure 5:**
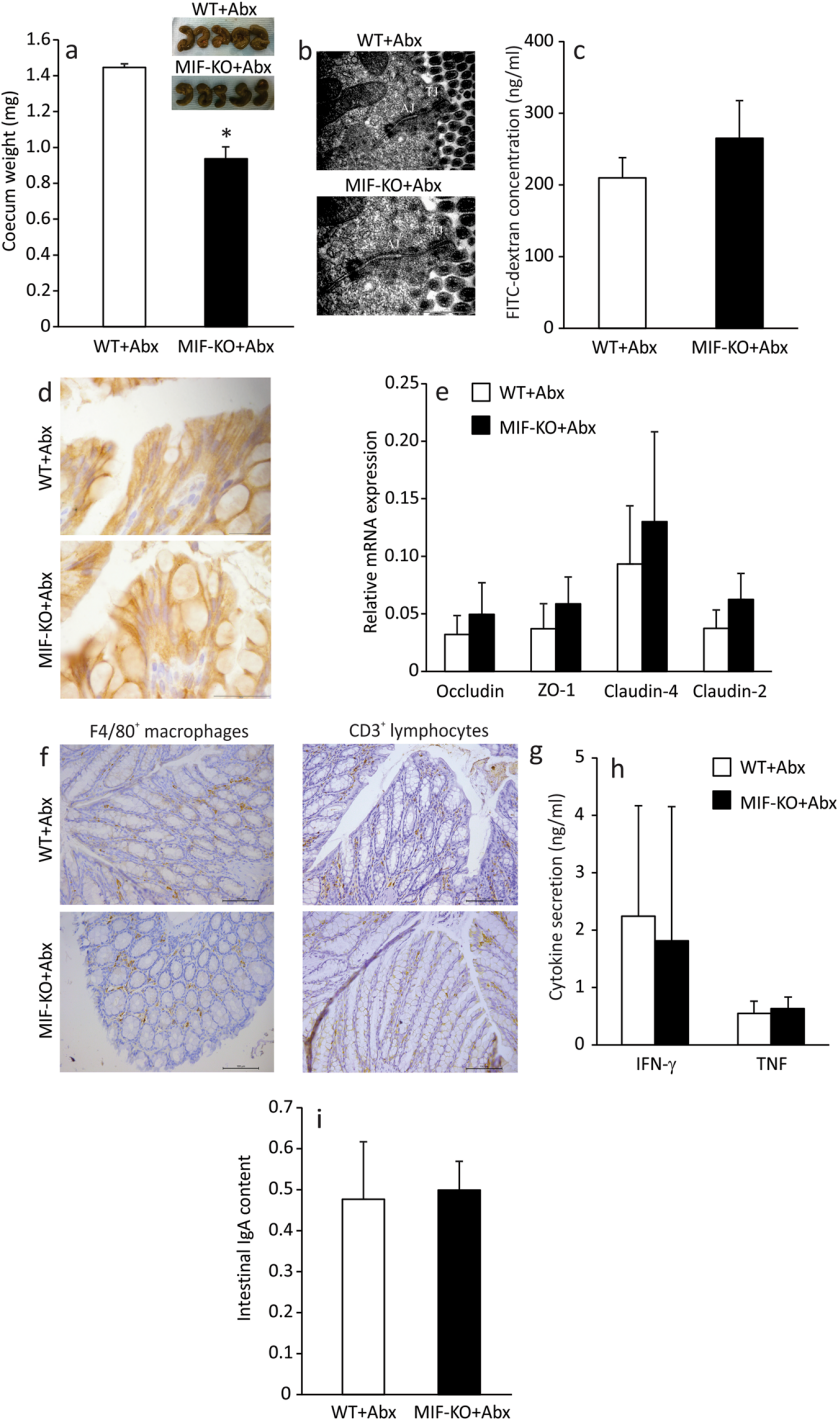
Modulation of microbiota improves intestinal barrier function and changes the GALT immune profile of MIF-KO mice. Caecum weight after the treatment with ampicillin and neomycin (Abx) with representative images above the graph (**a**). Electron micrographs of WT and MIF-KO colon sections (after the treatment with antibiotics) showing tight junctions and adherens junctions. 50 sections per mouse were analysed (**b**). Intestinal permeability after the antibiotic treatment measured by the presence of FITC-dextran in the serum (**c**). E-cadherin immunostaining on colon sections – representative images from at least 15 sections per mouse (**d**). Relative mRNA expression of tight junction proteins (**e**). F4/80^+^ macrophages (**f**) and CD3^+^ lymphocytes (**g**) in immunohistochemical sections of colon (lamina propria) after the treatment with antibiotics. At least 15 sections per mouse were analysed in a blinded fashion. Cytokine secretion in MLN after the treatment with antibiotics (**h**). Colon IgA content after the treatment with antibiotics (**i**). Groups consisted of 16 mice and graphs represent average values of all examined animals from 3 separate experiments. For statistical analysis, test of normality and Mann–Whitney U-test were used. *p<0.05 represents the significant difference between values obtained from WT vs MIF-KO mice.

As already described, a functional intestinal barrier consists of the epithelial lining, immune cells within the epithelium and certain microbiota at the luminal side of the barrier. Having in mind all the results of this study, it was still unclear which participant of the intestinal barrier is primarily affected by MIF. In order to investigate this, a change in microbiota content was introduced in MIF-KO mice by treating the mice with antibiotics (ampicillin+neomycin) through drinking water for 14 days. The antibiotic treatment increased the caecum weight (Fig. 5a) and caused a microbiota disturbance in both WT and MIF-KO groups (Fig. 4b–e) in more or less the same manner. Particularly, lactobacilli representing the core microbiota in WT and MIF-KO mice before the antibiotic treatment were replaced by *Enterococcus gallinarum*. However, the average diameter of tight and adherens junctions in between epithelial cells of MIF-KO mice was significantly lower (p = 0) compared to the level observed before the treatment with antibiotics (Table 1 and Fig. 5b). This was also confirmed through analysing the serum concentration of FITC-dextran after per os administration. After the antibiotic treatment, FITC-dextran serum concentrations of MIF-KO mice were more similar to those of WT mice, suggesting better control of intestinal permeability (Fig. 5c). This was accompanied by an increase in E-cadherin presence (Fig. 5d) and occludin expression (Fig. 5e) that probably restored the architecture of the epithelial junctions in MIF-KO. Interestingly, the expression of all examined tight junction proteins was considerably up-regulated in both WT and MIF-KO mice, which probably indicates the physiological response to the antibiotic insult (Fig. 5e). This is corroborated with the results of the recent study showing that a 7-day neomycin application increases mRNA expression of ZO-1, claudin-3 and claudin-4 in the colon^30^. Although antibiotics provoked growth of pathogenic or opportunistic pathogenic bacteria in MIF-KO mice, such as *Streptococcus pneumoniae*^31^, *Mycoplasma falconis*^32^, *Streptococcus oligofermentans*^33^, *Clostridium innocuum*^34^, *Klebsiella oxytoca*^34^, the number of macrophages (Table 2, Fig. 5f) and lymphocytes (Table 2, Fig. 5g) remained the same as before the treatment, while the levels of pro-inflammatory cytokines TNF and IFN-γ were reduced to the ones observed in MLN of WT mice under the antibiotic treatment (Fig. 5h). As for IgA levels, they did not change upon treatment with antibiotics (Fig. 5i). Seemingly, GALT of MIF-KO mice did not recognize this change in microbiota species as a danger signal, as it did before the antibiotic treatment.

The relationship between MIF on one side and gut microbiota, epithelial barrier and gut immune system on the other side is a very complex one. Having in mind that forced microbiota alteration prevented barrier “leakage” we propose the following scenario for the increased intestinal permeability in the MIF absence. The lack of MIF provokes improper immune surveillance in GALT that favours the growth of new microbiota species that may secrete metabolites which make the barrier more permeable. In conclusion, by affecting surveillance and composition of gut microbiota, MIF indirectly regulates the permeability of the intestinal barrier.

## Methods

### Animals

C57BL/6 (WT) and MIF-KO mice (bred on C57BL/6 background) were kept under standard conditions at the Animal Facility of the Institute for Biological Research “Sinisa Stankovic”. Male 2–6 months old mice were housed in the same room, exposed to the same environment, and used for the experiments. All experiments were approved by the Ethical Committee of the Institute for Biological Research “Sinisa Stankovic” (No: 02–07/16) and were in accordance with Directive 2010/63/EU. The mice were kept under standard conditions (12 h light/dark cycles) with free access to standard pelleted diet and tap water.

### Treatment with antibiotics

Both strains (3 months of age) were treated with Pentrexyl (ampicillin) and neomycin (Galenika A.D. Belgrade, Serbia) for 14 days. Antibiotics (1 mg/ml) were given through drinking water (5 ml a day for each mouse). *Ex vivo* analysis was performed one day after the treatment was seized.

### Isolation of immune cells

Immune cells were isolated from MLN which were removed aseptically and passed through a sterile plastic mesh (pores of 40 μm) in PBS + 3% FCS. After centrifugation, cells were counted and resuspended in RPMI 1640 supplemented with Antibiotic/antimicotic solution and 5% FCS (all purchased from PAA, Pashing, Austria). Cells were used either for obtaining conditioned supernatants (plated in 24-well plate for 24 h with or without concanavalin A – (ConA) – 10^7^ cells/ml), or placed in TriReagent (Fermentas, Vilnius, Lithuania) for obtaining protein and RNA fractions (5 × 10^6^ cells per sample).

### Isolation of gut epithelial cells

Small and large intestine were removed aseptically, cut into pieces (approx. 3 cm long) and cut longitudinally. After removing the intestinal content, pieces were washed thoroughly in cold PBS, transferred to dissociating solution (2.7 mM KCl, 150 mM NaCl, 1.2 mM KH_2_PO_4_, 680 mM Na_2_HPO_4_, 1.5 mM EDTA, 0.5 mM DTT) and left for 15 minutes on ice. In order to detach loosened cells, the pieces were vortexed for 15 seconds and solution with cells was collected. After repeating this procedure on remaining intestinal pieces for three times, the suspension was centrifuged at 1000 g for 10 minutes and resuspended in the TriReagent. The purity of epithelial cells (>94%) was determined indirectly by staining cell samples with immune cell-related antibodies (1.5 ± 0.3% CD3^+^ lymphocytes, 2.3 ± 1.3% B220^+^ B lymphocytes, 0.6 ± 0.3% F4/80^+^ macrophages and 2.0 ± 0.4% CD11c^+^ dendritic cells).

### Measurement of intestinal permeability

After overnight fasting, mice were administered with 50 μl of FITC-dextran (44 mg/100 g body weight) (Sigma-Aldrich, St. Louis, MO, USA) by oral gavage with a needle attached to a 1 ml syringe. After 4 hours, mice were bled from the retroorbital sinus and the intensity of the FITC dye was measured from obtained serum using Cameleon (Hidex, Turku, Finland) (excitation 488 nm, emission 534 nm). The measured values obtained from the sera without FITC-dextran (background fluorescence) were subtracted from the fluorescence intensity measured in the sera of treated mice. The exact concentration of FITC-dextran in the serum was calculated according to the standard curve values.

### Transmission electron microscopy (TEM) examination and ultrastructural morphometry

For electron microscopy, colon tissue samples from 5 MIF-KO and 4 WT animals were quickly immersed in 3% glutaraldehyde in cacodylate buffer and postfixed in 1% OsO_4_. After dehydration in graded alcohols, cells were embedded in Epoxy medium (Sigma-Aldrich). The ultrathin sections were stained with uranyl acetate and lead citrate and were examined with a Transmission Electron Microscope (TEM) (Morgagni 268D FEI, Hillsboro, OR, USA). Ultrastructural morphometry was performed on TEM ultra-micrographs on 50 tight and adherens junctions per tissue sample using the open-source software Fiji^35^. Width of adherens and tight junctions was represented by median in the interquartile range (IQR). IQR is a measure of statistical dispersion, being equal to the difference between 75th and 25th percentiles, or between upper and lower quartiles.

### Immunohistochemistry

Immunohistochemical analysis was performed on formalin-fixed, paraffin-embedded sections using following antibodies and dilution ratios: rat monoclonal anti-mouse F4/80 antibody (1:250, Abcam, Cambridge, UK), rabbit polyclonal anti-mouse CD3 antibody (1:500, Abcam), and rat monoclonal anti-mouse E-cadherin (1:200, eBioscience). Briefly, after dewaxing and rehydration, a heat-inducing antigen retrieval procedure using citrate buffer at pH 6.0 for 21 min was performed on all tissue sections, with subsequent washing in PBS and endogenous peroxidase blocking with EnVisionTM FLEX Peroxidase-Blocking Reagent (Dako, Agilent, Santa Clara, CA, USA) for 10 min. Sections were incubated with primary antibodies overnight. Sections stained with anti-F4/80 and anti-E-cadherin antibody were incubated with secondary polyclonal rabbit anti-rat immunoglobulins/HRP (1:500, Dako) for 60 min. The CD3 sections were treated by applying the commercial EnVisionTM FLEX/HRP detection reagent (Dako). Immunoreactions were developed with diaminobenzidine (DAB, Dako) diluted in EnVisionTM FLEX Substarte Buffer (Dako). The sections were counterstained with haematoxylin.

### Western blot

Proteins were isolated from the protein layer after the samples dissolved in TriReagent were centrifuged with chloroform at 12000 g following manufacturer’s instructions. Protein concentration was measured using Lowry assay as previously described^36^ and equal amounts of protein were layered onto acrylamide/bis-acrylamide gel (concentration of the gel was dependent on the molecular weight of the target protein). The immunoblot procedure was performed as previously described^37^. Secondary antibodies for anti-mouse IL-1β (1:1000, eBioscience) were FITC conjugated anti-armenian and syrian hamster IgG cocktail (1:1000, BD Bioscience, Bedford, USA) and HRP conjugated anti-mouse IgG (1:5000, Invitrogen, Carlsbad, CA, USA), for anti-mouse E-cadherin (1:200, eBioscience) it was HRP conjugated anti-rat IgG (1:5000, eBioscience), for anti-mouse IL-18 (1:800, Santa Cruz Biotechnology, San Diego, USA) it was HRP conjugated anti-chicken IgY (1:500, Thermo Fisher Scientific, Waltham, MA, USA), for anti-mouse biotin IgA (1:750; Invitrogen) it was avidin-HRP (1:500; eBioscience), and for anti-mouse tubulin (1:1000, Abcam), it was HRP conjugated anti-rabbit IgG (1:1000, Invitrogen).

### Measurement of IgA concentration in colon

Colon content (100 mg) was dissolved in 1 ml of PBS, vortexed for 5 min at room temperature and centrifuged (3000 g, 10 min). Supernatants were collected and 1 mM PMSF and 2 mM EDTA were added to prevent proteolysis. Samples were then vortexed and stored at −20 °C. IgA was detected by Western blot analysis and the final level of IgA was calculated as the ratio between the signal on the film and the level of total proteins in the supernatants measured by Lowry assay.

### RNA isolation, reverse transcription and PCR

Total RNA was isolated from the aqueous layer after the samples dissolved in TriReagent were centrifuged with chloroform at 12000 g following manufacturer’s instructions. RNA (1 µg) was reverse transcribed using RevertAid™ M-MuLV Reverse Transcriptase and random hexamer primers (Fermentas, Vilnius, Lithuania). PCR amplification of cDNA was carried out in Real-time PCR machine using SYBRGreen PCR master mix (Applied Biosystems) as described^37^. Primer pair sequences for β-actin were 5′-GACCTGACAGACTACC-3′ and 5′-GGCATAGAGGTCTTTACGG-3′ (NM_007393.2), for claudin-2 5′-TTAGCCCTGACCGAGAAAGA-3′ and 5′-AAAGGACCTCTCTGGTGCTG-3′ (NM_016675.4), for claudin-4 5′-AGCAAACGTCCACTGTCCTT-3′ and 5′-AATCCACCTCCACCCTTCTT-3′ (NM_009903.2), for occludin 5′-ACTACCTTGGGTGCTGTGCT-3′ and 5′-AAATTGGGCTGGATGTCAAT-3′ (NM_008756.2), and for ZO-1 5′-TCTTCCATCATTTCGCTGTG-3′ and 5′-TGTACATGCGTCCTGAAAGC-3′ (NM_009386.2). Gene expression was calculated as 2^−(Cti-Cta)^ (C_ti_ - cycle threshold of the gene of interest, C_ta_ - β-actin cycle threshold) and displayed as mean ± SD.

### Flow cytometry

Cells of MLN (5 × 10^5^) were washed in PBS + 1% BSA and stained with anti-mouse CD3-PE, CD4 FITC, CD8 PE, F4/80 FITC, CD40 PE, CD206 PE, CD25 PE, CD11c PE-Cy5, NK FITC, B220 FITC antibodies (eBioscience) in FlowCytometry Staining Buffer (eBioscience) for 1 h at 4 °C in the dark. After repeated washing in PBS + 1%BSA, cells were resuspended in PBS and immediately detected by CyFlow Space (Partec, Görlitz, Germany) and analysed by FlowMax software (Partec). Prior to intracellular cytokine staining, cells (1 × 10^6^) were stimulated with phorbol myristate acetate (PMA, 100 ng/ml) and ionomycin (400 ng/ml) (both from Sigma-Aldrich) in the presence of Brefeldin A (eBioscience) (5 µM) for 4 h, stained with anti-mouse CD5 FITC and anti-mouse CD19 PE-Cy5 (eBioscience) antibody, fixed in 2% paraformaldehyde, permeabilized with Permeabilization buffer (eBioscience) and then stained for the intracellular cytokines with the anti-mouse IL-10 PE (eBioscience).

### Viability assay

MLN cells (10^7^/ml) were treated with 300 μl of 0.5 mg/ml 3-(4,5-dimethyl-2-thiazolyl)-2,5-diphenyl-2H-tetrazolium bromide (MTT) solution (Sigma-Aldrich) for 30 min at 37 °C in a humidified incubator. After the transformation from tetrazolium salts to formazan crystals in viable cells, dye in the cells was dissolved with DMSO and measured by LKB microplate reader (LKB Instruments, Vienna, Austria) at 540 and 670 nm.

### Measurement of cytokine levels

Cytokine concentration in cell culture supernatants was determined by sandwich ELISA using MaxiSorp plates (Nunck, Rochild, Denmark) and anti-mouse paired antibodies according to the manufacturer’s instructions. Samples were analysed in duplicate for murine TNF, IL-6, IL-1β, IFN-γ, IL-17, IL-4, and TGF-β (eBioscience) and absorbance was measured by LKB microplate reader at 450 and 570 nm. A standard curve created from the known concentrations of appropriate recombinant cytokines was used to calculate concentration values of measured cytokines.

### Denaturing Gradient Gel Electrophoresis (DGGE) analysis and DNA sequencing

Extraction of bacterial DNA from frozen faecal samples was done using the QIAamp DNA stool minikit (Qiagen, Hilden, Germany). DGGE analysis and gel manipulation after electrophoresis was entirely performed as described previously^38^. Primer sets complementary to 16S rDNA, specific for Lactobacilli (Lab-0159f paired with the universal reverse primer Uni-0515-GCr^39^) and for Eubacteria (U-968-GC-f pared with L1401-r^40^) were used. Fragments of interest were excised from the gel and macerated, and the suspension was incubated for 10 min at 98 °C^39^. After incubation, the suspension was centrifuged to pellet gel particles. The supernatant (30 ml) was used in PCRs with Lab-0159f and Uni-0515GCr primers^39^ and U-968-GC-f and L1401-r^40^. The obtained PCR products were purified using the QIAquick PCR purification kit (Qiagen) and ligated into the pBluescriptT/A vector^41^. Ligated constructs were transformed in Ca^2+^-induced competent DH5α cells^42^, and insert-containing transformants were selected as white colonies on Luria agar (LA) plates containing 100 mg/ml ampicillin and 20 mg/ml X-Gal (5-bromo-4-chloro-3-indolyl- b-D-galactoside) as recommended by Promega. For each excised DNA band, one white colony was picked and plasmids were isolated using the QIAprep spin miniprep kit (Qiagen). The sequencing of the isolated insert-containing pBluescriptT/A plasmids was done with M13F/R primers at Macrogen Europe Service, Amsterdam, Netherlands (http://dna.macrogen.com/eng/support/ces/guide/universal_primer.jsp). Sequence annotation and the database searches for sequence similarities were performed with the BLAST tool available online (https://blast.ncbi.nlm.nih.gov/Blast.cgi).

### Statistical analysis

Data are presented as mean ± SD or median and interquartile range (25th to 75th percentile). Statistical analysis was performed using Statistica 6.0 (StatSoft Inc., Tulsa, USA) software. Comparisons between the groups were done by 1-way ANOVA, followed by Student–Newman–Keuls post hoc test and finally Student’s t-test or Mann–Whitney U-test where appropriate. *p* value less than 0.05 was considered to be statistically significant.

### Data availability statement

The datasets generated during and/or analysed during the current study are available from the corresponding author on reasonable request.

## Electronic supplementary material

Supplementary data
