# Supplementary material for: The Role of Macrophage Migration Inhibitory Factor in the Function of Intestinal Barrier
Source: Sci Rep. 2018 Apr 20;8:6337. doi: 10.1038/s41598-018-24706-3 (PMC5910418; doi:10.1038/s41598-018-24706-3)

The role of macrophage migration inhibitory factor in the function of intestinal barrier

MilicaVujicic^1#^, Tamara Saksida^1#^, Sanja Despotovic^2^, Svetlana Sokovic Bajic^3^, Ivana Lalić^2^, Ivan Koprivica^1^, Dragica Gajic^1^, Natasa Golic^3^, Maja Tolinacki^3^, Ivana Stojanovic^1*^

^1^Institute for Biological Research “Sinisa Stankovic”, University of Belgrade, Department of Immunology, Belgrade, 11060, Serbia

^2^Faculty of Medicine, University of Belgrade, Institute of Histology and Embryology, Belgrade, 11000, Serbia

^3^Institute of Molecular Genetics and Genetic Engineering (IMGGE), University of Belgrade, Belgrade, 11000, Serbia

Supplemental data

Histopathological examination of the colon

Colon tissue samples (5 MIF-KO and 4 WT animals) were fixed in 10% neutral buffered formalin, processed to paraplast and tissue sections (4-5 μm thick) were routinely stained with hematoxylin and eosin. Microphotographs were acquired with digital camera Leica DFC295 (Leica, Heerbrugg, Switzerland) connected to a light microscope Leica DM4000 B LED (Leica, Wetzlar, Germany). Measurements were performed on 3 sections per animal by using open-source software Fiji^27^. All elements of intestinal mucosa were measured. The thickness (in μm) of intestinal mucosa, submucosa and tunica muscularis were measured (3 measurements on each section), the thickness of lamina muscularis mucosae (3 measurements on each section), as well as the crypt depth (10 measurements on each section) using longitudinally sectioned Lieberkühn crypts at x200 magnification. The height of surface epithelium (3 measurements on each section) was measured at x400 magnification. Crypt diameter, crypt perimeter and the height of crypt epithelium were measure at x400 magnification on tissue samples with cross-sectioned crypts, on 10 crypts per section. Also, the number of nucleated cells in the lamina propria (per 0.1 mm^2^ of tissue) was measured on cross-sections of intestinal lamina propria at x400 magnification on 3 measurements on each section.


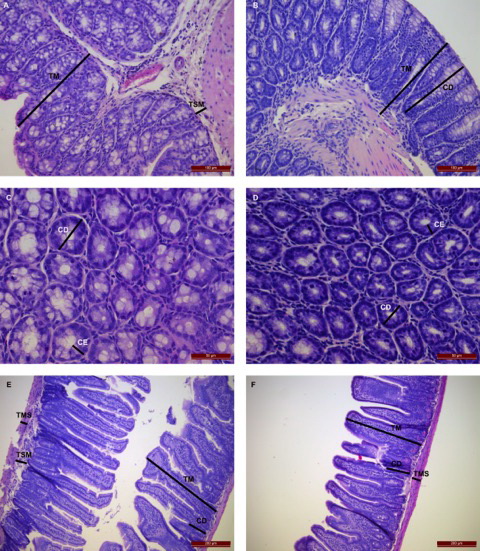


Fig. S1. Intestinal architecture in the colon and small intestine. HE-stained sections from colon of WT (A, C, E) and MIF-KO animals (B, D, F). TM-tunica mucosa, TSM- tunica submucosa, TMS- tunica muscularis, CD- crypth depth, CE- cripth epithelium, CD- crypth diameter;

| Colon | WT  (average ± standard deviation) | MIF-KO  (average ± standard deviation) |
| --- | --- | --- |
| Thickness of tunica mucosa (TM) in μm | 262.25 ± 53.64 | 282.94 ± 64.27 |
| Thickness of tunica submucosa (TSM) in μm | 20.32 ± 10.07 | 17.56 ± 7.68 |
| Thickness of tunica muscularis (TMS) in μm | 75.89 ± 30.71 | 80.68 ± 36.83 |
| Thickness of lamina muscularis mucosae (LMM) in μm | 13.49 ± 4.81 | 11.56 ± 4.22 |
| Height of surface epithelium in μm | 18.99 ± 4.07 | 16.29 ± 3.14 |
| Crypth depth in μm | 200.65 ± 40.99 | 220.98 ± 40.83 |
| Height of crypt epithelium in μm | 18.46 ± 6.51 | 14.13 ± 2.12 |
| Crypt diameter in μm | 49.32 ± 18.67 | 52.58 ± 4.39 |
| Crypt perimeter in μm | 152.37 ± 25.34 | 154.76 ± 4.91 |
| Number of cells in the lamina propria (per 0.1 mm^2^ of tissue) | 1110.04 ± 294.18 | 1013.34 ± 134.61 |

Table S1. Elements of colon intestinal mucosa.

Figure S2. Gel image that has been flipped on the horizontal axis to match figure legend in Figure 2A in the manuscript.





Figure S3. Gel image that has been flipped on the horizontal axis to match figure legend in Figure 2C in the manuscript.





Figure S4. Additional DGGE gels with *Lactobacillus* primers.


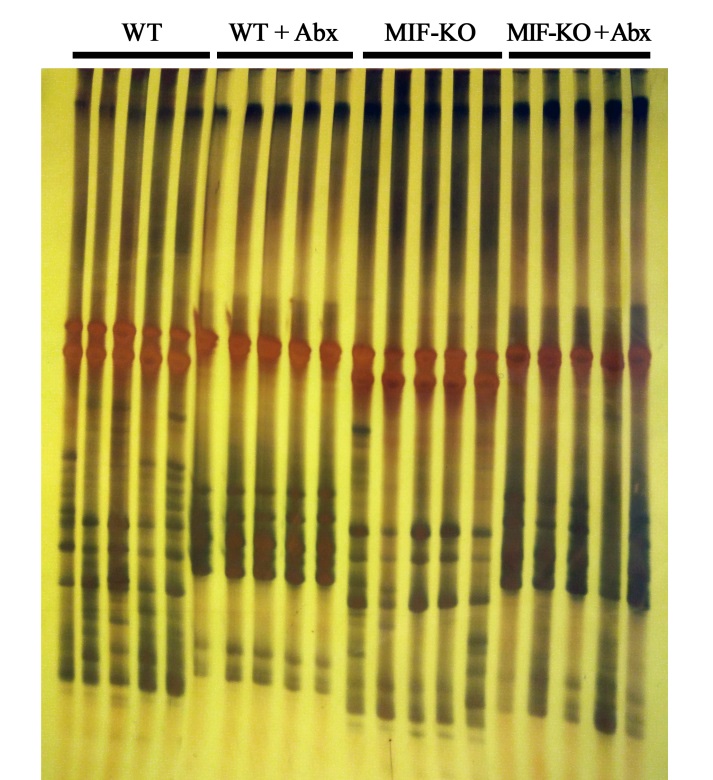


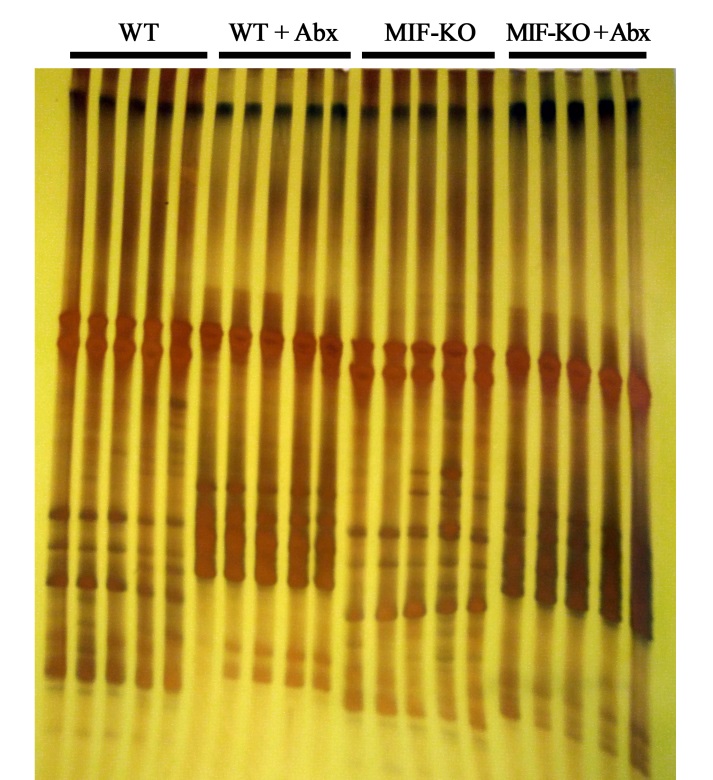


Figure S5. Additional DGGE gels with universal bacterial primers.


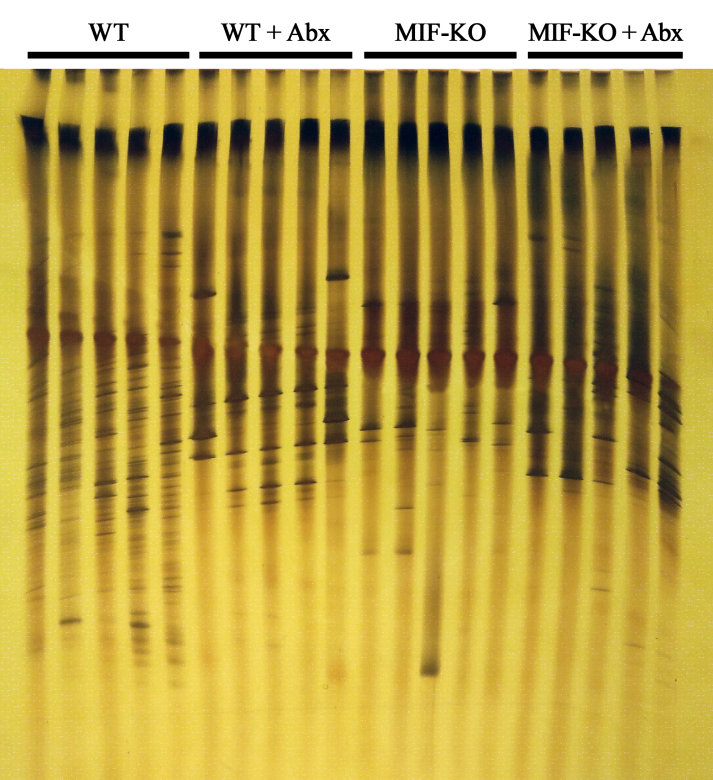


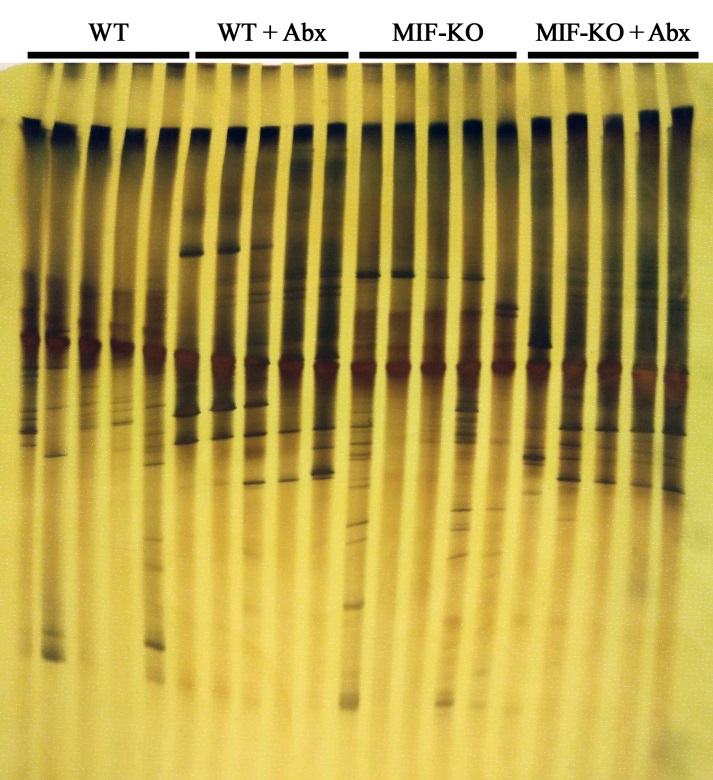

Supplement: Supplementary file 1 — Supplementary data [file 41598_2018_24706_MOESM1_ESM.docx]
